# Declining Caries Trends: Are We Satisfied?

**DOI:** 10.1007/s40496-015-0064-9

**Published:** 2015-09-23

**Authors:** M. D. Lagerweij, C. van Loveren

**Affiliations:** Department of Cariology Endodontology Pedodontology, ACTA, Amsterdam, Gustav Mahlerlaan 3004, 1081 LA Amsterdam, The Netherlands

**Keywords:** Caries, Epidemiology, Socio-economic status, Trend, Prevention, Fluoride

## Abstract

WHO data suggest that all over the world the prevalence of caries has declined at the end of the previous and in the first decade of the present century. This decline started wherever the use of effective fluoride toothpaste became commonplace. Even though the decline is considerable with a 90 % reduction in DMFT for 12-year-olds in Western Europe and the USA, caries still affects 60–90 % of the children throughout the world. In the high- and middle-income countries, the nature of caries has changed from a rapid progressing disease of childhood to a slowly progressing disease throughout adulthood and even old age. However, throughout the world, the circumstances for caries differ, e.g., low-income countries experience more caries with higher sugar consumption, while between high-income countries this correlation is reversed. In high-income countries, fluoride is widely used and preventive programs in dental offices are in place. These programs, if effective, may not be a realistic option in low-income countries. In order to reduce caries in the world even further, the use of effective and affordable fluoride toothpaste should be encouraged and enabled.

## Introduction

Worldwide data on caries prevalence are scarce and moderately reliable. WHO collected data for the indicator group of 12-year-olds in the WHO’s Oral Health Country/Area Profile Programme (CAPP) database [1•]. Comparison between the countries, however, is difficult because of the variance in the internal and external validity of the studies and in the years of data collection. Caries prevalence is often reported in the form of the DMFT or DMFS value of the population, where D stands for decayed, M for missing due to caries, F for filled due to caries, and T means that the data are reported with one value per tooth, while S means reported one value per tooth-surface. As caries can be scored at various levels of severity ranging from white spot lesions to frank cavitation, the D may be noted with a suffix indicating at which level of severity caries is reported. D_1_ includes all forms of caries while D_3_ only includes frank cavitation or more severe forms [2]. From data collected after 2000, the highest caries levels can be seen in the Latin American countries (D_3_MFT 2.4 ± 1.2) and in Europe (D_3_MFT 2.1 ± 1.2; Western Europe D_3_MFT 1.0. ± 0.2; Eastern Europe D_3_MFT 3.2 ± 0.8), while lower figures are seen in the Middle East (D_3_MFT 1.9 ± 1.4), the Western Pacific (D_3_MFT 1.5 ± 1.0), in Southeast Asia (D_3_MFT 1.2 ± 1.2), Africa (D_3_MFT 1.2 ± 1.6), and North America (D_3_MFT 1.1 ± 0.1). Within each WHO region, there are large differences between countries, for instance, in Africa, Gabon stands out with D_3_MFT 4.9; in Latin America, Guatemala (D_3_MFT 5.2), Peru (D_3_MFT 3.7), and Panama (D_3_MFT 3.6); in the Middle East, Saudi Arabia (D_3_MFT 5.9) and Lebanon (D_3_MFT 3.4); in South Asia, in India (D_3_MFT 3.9), and in the Western Pacific, Cambodia (D_3_MFT 3.5) and the Philippines (D_3_MFT 3.3).

The WHO data suggest that all over the world the prevalence has been declining at the end of the previous and during the first decade of the present century. The largest decline is seen in the high- and middle-income countries while in the low-income countries, the decline is less explicit. There are a few exceptions where caries prevalence has increased, e.g., Gambia, Saudi Arabia, Moldova, and Croatia [1•]. The decline was first observed in the USA and Western and Nordic European countries. In these forerunner countries, the decline started in the mid-1970s of the previous century after effective fluoride toothpaste became widely used [3–5] reaching a 90 % reduction in the mid-1990s of the previous century resulting in a decrease from approximately D_3_MFT 8 to approximately 1 [6]. In Eastern Europe, the decline only started after the fall of the iron curtain when the market was opened for effective fluoride toothpaste. This explains partially why caries prevalence is still significantly higher in the eastern parts of Europe (D_3_MFT 3.2 ± 0.8) as compared to the western parts (D_3_MFT 1.0. ± 0.2). The rapid unification of former West and East Germany resulted in a rapid evenness in oral health. The D_3_MFT number for 12-year-olds in East Germany declined from 5.7 in 1981 to 2.6 in 1997 and 1.1 in 2005, while in West Germany, this decline was 5.1, 1.4, and 0.7, respectively [7, 8].

The caries decline is also manifest in younger adult and middle-aged population groups. This is illustrated when comparing four cross-sectional epidemiological studies performed in 1973, 1983, 1993, and 2003 in Jönköping, Sweden, among individuals aged 20, 30, 40, 50, 60, 70, and 80 years (Table 1). When comparing the D_1_FS of 20- and 30-year-olds at the various time points, it can be seen that both the prevalence of the 20-years-olds decreased and the increment between the two age cohorts decreased, but most of the caries of each age cohort developed before the age of 20 [9].

**Table 1:** Mean number of D_1_FS in 1973, 1983, 1993, and 2003 in Jönköping, Sweden [9]

Age group (years)	Mean number of D_1_FS			
	1973	1983	1993	2003
20	35.1	21.5	15.7	9.7
30	48.4	40.7	23.3	14.0
40	52.6	53.0	41.2	23.3
50	50.5	53.6	55.2	37.0
60	44.5	46.2	53.2	52.5
70	41.0	39.1	52.4	51.0
80		34.4	45.2	53.8

In the forerunner countries, the reduction seemed to have reached a plateau in the younger age groups. The early birth cohorts with apparently a maximum obtainable effect get older, and at older age groups, the decline can still be observed till the birth cohorts with that maximum effect reach that specific age. In some forerunner countries, there are signals that caries prevalence of 5- and 6-year-olds is showing a slight increase emphasizing the necessity not to relax in the preventive efforts [10, 11].

## Explanation for the Decline

This decline of caries prevalence has been mainly attributed to the effective introduction of fluoride in toothpaste and the awareness and commitment of people to maintain high level of oral hygiene throughout life [12]. Interestingly, the consumption of sugar has been steadily increasing or remained stable even in the period where caries prevalence had been declining [13, 14]. Obviously, when fluoride is used, the threshold below which sugar can be used safely has increased. Duggal et al. showed this phenomenon elegantly in an in situ model [15]. When the subjects used a fluoride-containing toothpaste, demineralization only occurred after more than 7 sucrose exposures/day, while during the use of fluoride-free toothpaste, the threshold was at 3 exposures/day.

In areas where fluoride is not or insufficiently used, regular sugar intake is, however, still a major caries risk. From ecological data on the relationship between DMF and sugar consumption [16] and from the effect of the wartime diets on caries prevalence [17], Sheiham [18] suggested that at and below approximately 15 kg sugar/person/year, most of the population will not develop dental caries. This amount corresponds with the 10E% as recently proposed by the WHO to limit the consumption of free sugars to [19, 20]. For many countries, this would imply over 50 % reduction in the sugar consumption. It can be questioned whether this is a realistic goal, because it would require a significant change in dietary habits of the people and significant change in production policies of the nutritional industry to reduce the sugar content of their products. Awaiting for these developments if they are realistic at all, it is important to continue to emphasize the importance of the reduction of the frequency of intake as being an important item in dietary counseling [21]. When counseling, however, the paramount importance of the twice daily use of fluoride toothpaste should always be stressed.

## Limitation of the Data

The data of the 12-year-olds may suggest that worldwide caries is only moderately present [22]. This is not the case. In fact, the WHO reports that dental caries is the most common childhood disease and non-communicable disease worldwide [23]. 60–90 % of the children are affected and mostly untreated due to inappropriate, unaffordable, or unavailable oral health care services. In a high-income country as the Netherlands, most of the health resources for children are spent on dental caries [24, 25]. The average DMFT of 12-year-olds is, therefore, not a very reliable criterion for a conclusion on the total health burden of dental caries. At the age of 12, only a few permanent teeth are erupted. The teeth that are erupted are only relatively shortly at risk in the mouth. An average figure does not do justice to the fact that the distribution is skewed. D_3_MFT does not capture pre-cavitated lesions nor does it indicate severity in terms of pulp involvement or odontogenic infections. Furthermore, the 12-year-olds may benefit more from collective preventive measures than other age groups, for instance, preschool children or care-dependent people.

The D_3_MFT does not give an indication of the level of care. This implies that even in countries where D_3_MFT is low, there may be a significant amount of untreated caries. Van Palenstein et al. [26••] reports on the D_3_, M, and F component separately for higher income, upper middle income, lower middle income, and low income. While in the high-income countries, almost 50 % of the caries is treated; in the lower income countries, this proportion only accounts for 2 % of the caries [1•, 26••].

## Caries Progression Rate

It has been suggested that the nature of primary caries has changed from a rapidly progressing disease of childhood to a slowly progressing disease which commences in childhood but progresses steadily in adulthood [27]. Till the age of 13, fissure caries constitutes approximately 80 % of the caries, while at the age of around 25, the amount of approximal caries surpasses the amount of fissure caries [28]. As the mean caries experience declines in the population, the progression rate through enamel decreases [29]. The rate of caries progression depends on age, maturation of the enamel, localization on the tooth within the dentition, the depth of the lesion, and the caries risk. But how long does it take for a lesion to progress through the enamel? This is not an easy question to answer, because there are only a few longitudinal studies published and they are difficult to compare. Most data on the caries progression rate come from Röntgen image studies on the approximal surfaces. Schwartz found in US 10-year-old children, which were at high caries risk, that it took on average 22 and 19 months for caries to progress through, respectively, the outer half and inner half of the enamel [30]. In the Swedish children, regarded as low-risk children, the progression rates were 22 and 28 months, respectively. At the age of 17 of the US children, the progression rate through the outer half of the enamel was 16 months and through the inner half 27 months and for the Swedish children, respectively, 40 and 50 months. Lervik et al. [31] studied the x-rays of 65 patients aged 15 till 19 years and estimated the caries progression rate through enamel to be 6 years. Brabner et al. [32] found in 12-year-olds with a high risk of caries on the Isle of Wight that 12 % of the initial lesions had progressed through the enamel to the dentine within a year, 46 % within 2 years and 62 % within 3 years. Mejàre [28] found in age group 11 till 26 years that initial enamel lesions took 4.2 years to progress to the dentine, where this rate varied throughout the mouth between 2 years for the molars and 5 years for the lower premolars.

## Caries from Epidemic to Endemic

In the pre-fluoride era (sixties and seventies of the previous century) caries in the western world could be described as an epidemic disease. Being caries free was virtually non-existent and caries would often have affected every tooth by the age of 18. There were hardly any sites that could become affected after the age of 18. Caries was an epidemic disease. Nowadays, caries can be described being endemic with large groups of population with little or moderate caries and a small group with high levels of caries. This was nicely shown in the Dunedin study where a large cohort was followed from the age 5 till the age of 38 [33••]. Approximately, 40 % of this cohort belongs to the low caries group, 45 % to the moderate caries group, and 15 % to the high caries group. The persons in each trajectory could already be identified at an early age (caries as a predictor for caries). Amazingly, the yearly caries increment remained stable towards the end of the 32-year period of observation. This indicates that caries nowadays cannot be seen as a disease for children only, but that caries risk continues throughout life.

Previously, many older people would have a full denture, but the contemporary and future generations of elderly keep more and more of their natural teeth as a result of the decline of caries [34]. Caries risk, however, may increase when people get older and their roots get exposed. Many elderly may receive multiple medication that reduces the quantity and quality of their saliva [35]. This will impose a risk to the oral health in two ways: less saliva means a reduced ability to neutralize the acids produced by cariogenic bacteria, and lower quality means a reduction in anti-microbial efficacy. In addition, older people may forget to brush, and their self-control to eat sweets may loosen. Lastly, the ability of elderly to maintain their oral hygiene can be seriously hampered by a reduction in manual dexterity necessary for brushing. Their oral health will be at serious risk and programs have to be developed to assist these people in their daily care [36, 37].

## Different Circumstances Different Problems

It would be wrong to look everywhere in the world to caries as the same problem with the same solution. For instance, the relationship between sugar consumption (kg/capita/year) with DMFT is directly proportional among low-income countries, but inversely proportional among high-income countries [38]. Between low-income countries, this relationship was stronger when there was a high-income inequality. The social gradient for oral health is different in high- and low-income countries [39•]. Where in high- and middle-income countries the disease is concentrated in the lower socio-economic strata, ecological observations suggests that in low-income countries affluence may increase the risk of caries by the use of cariogenic products (i.e., containing refined added sugar). The levels of care, and therefore professional support, differ throughout the world. Interestingly, however, there is only a weak association between the number of DMFT among children and the number of dentist per capita [22]. There are substantial differences in DMFT index scores among countries that have the same number of dentist per capita, indicating that many other factors affect dental health beyond the availability of dentists [22].

## Future Perspectives

Although the average D_3_MFT of 12-year-olds in various parts of the world is not that different, their future perspective is. In the western high-income world, all preventive measures are available and the general lifestyle trends and health literacy are towards health. In the low- and middle-income countries, these developments are less favorable. All low- and middle-income countries are undergoing major demographic, economic, technological, social transitions favoring caries risk factor exposure including higher consumption of tobacco, alcohol, salt, and sugar [25]. The composition of the diets changes from less-cariogenic complex carbohydrates to more refined prepared products. Lifestyle transitions that include increasing sugar consumption without appropriate fluoride exposure will increase the caries risk [25].

Dentistry is being pulled two ways: wealthy members of society demand high-end expensive treatment, much of it cosmetic rather than necessary to deal with disease, whereas many millions of poor people in developing countries cannot afford basic dental treatment and may never see a dentist. Too many governments and dentists persist with the expensive and destructive regime of “drill and fill (and bill).” Dentistry should move from an increasingly un-affordable curative model to a cost-effective evidence-based preventive model. The goal is to help people retain healthy natural teeth throughout their lives, as an essential part of enhancing their general health [40•].

## Not One Solution

It may be clear that the caries problem cannot be solved everywhere in the world in the same way, although everywhere in the world oral health may benefit from reducing the social inequalities and following the common risk strategy for diseases [41]. But, countries are too different with respect to their resources and infrastructure to advocate one solution. There may be one common pitfall: believing that the professional preventive measures can solve the problem. For the high-income countries, you can even wonder whether the effects of professional preventive measures are not overestimated. A program consisting of all thinkable measures was not effective in high-risk children [42]. The same program showed to be effective when the preventive program was individually patient-centered aimed at identifying and eliminating factors that had led to the presence of active caries. The program included counseling sessions with emphasis on enhancing use of the children’s own resources in everyday life [43]. These studies show that telling the patients what to do is not sufficient and that professional treatments cannot compensate this. Instead, patients and parents should be coached to learn the factors that lead to and protect against dental disease and be assisted in selecting self-management goals to improve their own and their children’s risk for disease [44•]. Key factors in these strategies are the appropriate use of fluoride toothpastes. The preventive programs are not necessarily done in a dental office or by a dental professional but can also be guidance for public dental health strategies.

## Conclusions

Caries is still the most widely spread non-communicable disease in the world. It may affect everybody throughout one’s lifetime and proper daily oral care is paramount. In high-income countries, the number of carious lesions in 12-year-olds is considerably reduced by 80–90 %. But the treatment of caries still consumes a high proportion of the health resources. In the moderate- and low-income countries, caries levels are seemingly on a level comparable to the high-income countries but the prospectives are completely divergent. Fluoride and proper oral hygiene, however, are the key components for every oral health program. Caries declined: are we satisfied? No, we believe that we still have a long and difficult way to go.
